# Generalization Effects in Evaluative Conditioning: Evidence for Attitude Transfer Effects from Single Exemplars to Social Categories

**DOI:** 10.3389/fpsyg.2017.00103

**Published:** 2017-01-31

**Authors:** Tina Glaser, Dieta Kuchenbrandt

**Affiliations:** Department of Psychology and Sport Sciences, Bielefeld UniversityBielefeld, Germany

**Keywords:** evaluative conditioning, generalization, attitude change, lateral attitude change, contingency awareness

## Abstract

The present research investigated whether evaluatively conditioned attitudes toward members of a social category (CSs) generalize to other stimuli belonging to the same category as the CSs (generalization at the stimulus level) and to the category itself (generalization at the category level). In four experiments, USs were paired with schematic or naturalistic CSs belonging to certain fictitious groups. Afterward, attitudes toward the CSs, toward non-presented exemplars of the CS category, and toward the CS category were assessed. Results revealed evidence for generalization effects in EC on both the stimulus and the category level. Transfer effects were greater when participants’ awareness of the CS–US contingency (CA) was high. Moreover, we found differences in generalization between the stimulus and category level, indicating that different processes might contribute to the effects. Theoretical and practical implications such as using EC as a tool for changing attitudes toward social groups will be discussed.

## Theoretical Background

In June 2016, we could read in German newspapers about Naja and Mouath, two Syrian refugees who helped the German inhabitants of the small town Simbach in Bavaria to clear away rubble resulting from a serious flooding that had swept through parts of Bavaria after heavy rainfalls. How does that information influence your attitude toward Naja and Mouath? It is likely that you will develop a positive attitude toward them. Now, what would happen if you meet Hamit, another refugee from Syria? Intuitively, you would probably also like Hamit. Your positive attitude toward Naja and Mouath would have generalized to Hamit because they share an important characteristic: They belong to the same national group. Such spreading of attitudes from one target person to another person or to the whole group could be a key mechanism underlying the formation of intergroup attitudes. Research on intergroup attitudes has shown repeatedly that positive intergroup interactions (direct but also indirect vicarious interactions) do not only improve attitudes toward the specific out-group member but also reduce negative bias toward the whole out-group (e.g., Pettigrew, 1998; Ensari and Miller, 2002; Pettigrew and Tropp, 2006) and even toward other groups (Pettigrew, 2009). Can such a generalization of attitudes from one attitude object to another or to the whole object category be understood in terms of evaluative conditioning (EC)?

Evaluative conditioning refers to changes in (dis-)liking that are caused by the pairing of stimuli (De Houwer, 2007). In a prototypical EC study, a subjectively neutral picture (conditioned stimulus; CS) is repeatedly presented with a subjectively (dis-)liked picture (unconditioned stimulus; US). The common result is that the formerly neutral CS acquires the evaluative quality of the US. The EC effect is quite robust and has been demonstrated in a large number of areas with different kinds of USs and CSs (for a meta-analysis see Hofmann et al., 2010). One important question in EC research is concerned with the mental processes underlying EC (for overview see Hofmann et al., 2010). Some EC accounts more or less explicitly focus on the formation of associations in memory between the cognitive representations of CS and US (e.g., Levey and Martin, 1975; Baeyens et al., 1992). Once an association is formed, the CS activates the liking that was originally evoked by the US. Other EC accounts emphasize the role of higher order mental processes. These propositional approaches assume that EC is based on the formation and truth evaluation of propositions about CS–US relations: The evaluation of the CS changes because people form a conscious proposition that the CS is paired with a positive or negative US (De Houwer, 2009; Mitchell et al., 2009). Although the formation of a proposition is considered to be non-automatic, the proposition can be stored in memory and retrieved automatically (Zanon et al., 2012). The question of the underlying processes is directly related to an ongoing debate regarding the role of contingency awareness (i.e., awareness of the CS–US pairings, CA) in EC, particularly whether EC is independent of, facilitated, or impeded by CA (e.g., Balas and Sweklej, 2012; Gawronski and Walther, 2012; Hütter and Sweldens, 2013). Assuming that EC is based on automatic associative processes, EC effects should occur independently from CA. In contrast, propositional accounts imply that CA is a prerequisite for EC effects. Although there is evidence for both contingency-aware (e.g., Pleyers et al., 2007) and contingency-unaware EC (e.g., Walther, 2002; Hütter and Sweldens, 2013), effects are usually larger when participants are aware of the CS–US contingencies (Hofmann et al., 2010). Thus, CA serves as an important moderator for EC.

To learn more about the potential role of EC for the formation or change of intergroup attitudes, it is essential to know whether EC effects generalize to other stimuli. We define generalization as the spreading or transfer of an attitude from the original target of attitude change to a related attitude object. Thus, in line with the Lateral Attitude Change (LAC) model (Glaser et al., 2015), generalization is defined as an effect (cf., De Houwer, 2007), whereby a change in the evaluation of a focal attitude object, in this case the CS, goes along with a change in evaluation of lateral attitude objects, in this case other stimuli of the CS category and the category itself. EC research so far has mainly concentrated on testing the outcome of the pairing procedure for the CS, and for the CS only. However, there is initial evidence that EC effects can transfer to other attitude objects not present during the conditioning procedure. Walther (2002) found that the EC procedure not only affected the evaluation of the CS but also “spread” to the stimulus that was experimentally pre-associated with the CS. Thus, the CS as well as the pre-associated stimulus was evaluated according to the valence of the US although only the CS was directly presented together with the US.

Hütter et al. (2013) focused directly on the generalizability of EC effects. Specifically, they successfully distinguished evaluative identity conditioning (i.e., conditioning of an individual CS) from evaluative cue conditioning (i.e., conditioning of a cue representing a social category such as age or gender). They did so in order to examine whether EC effects generalize to stimuli that share a specific cue value with the original CS. To illustrate, they paired CSs (faces) sharing a cue value ‘male gender’ (operationalized via short hair) with positive USs. However, a small minority of short hair CSs was paired with negative USs. As expected, they found that the positive conditioning of the cue ‘male gender’ resulted in positive evaluations of the short hair CSs. Importantly, this effect occurred even for the few short hair CSs that were not paired with positive but with negative USs. With this, the authors demonstrated that EC is not restricted to attitude change toward individual CSs but can also be obtained for social category cues. However, as stated by Hütter and colleagues (2013, footnote 6), evidence for transfer effects on stimuli that shared the same cue value but were *not* presented during conditioning was relatively unreliable across their three studies. Furthermore, the authors acknowledge that the conditions under which these kind of transfer effects can be observed were not covered in their research.

Recent research by Spruyt et al. (2014) showed that EC generalization depends on feature-specific attention allocation. Specifically, only generalization stimuli that were similar to the CS in terms of the stimulus dimension that was selectively attended to were evaluated in congruence with the valence of the respective CS. Moreover, significant generalization effects were obtained only for participants who had accurate (vs. inaccurate) CA. Different from Hütter et al. (2013), this research demonstrated generalization effects for stimuli that were not part of the conditioning procedure. However, generalization effects on the category itself were not investigated.

The only evidence for generalization within EC using ethnic social categories comes from Olson and Fazio (2006). In their experiments, they unobtrusively paired pictures of Black individuals with positive stimuli and pictures of White individuals with negative stimuli. In a control group, participants viewed the same stimuli without any pairings. As dependent variables, they used a priming measure of racial attitudes as well as explicit racism scales and a feeling thermometer. The authors report that participants in the experimental group showed less implicit racial bias than participants in the control condition. Although Olson and Fazio’s (2006) studies are an important step in providing evidence for an improvement of intergroup attitudes via EC, some critical issues need to be considered when interpreting these findings. First, in the control condition they found a positive bias in favor of Whites but no difference in reaction times for negative words. Moreover, this two-way interaction of race of prime and target valence was only marginally significant. In the experimental group, also no difference in reaction times to negative words was found. Instead, the authors report only a non-significant difference for positive words, showing a tendency that participants in the experimental group were faster in reacting to positive words when preceded by a Black versus White face. Second, on explicit measures, Olson and Fazio (2006) found no significant generalization effects; they even report a trend in a negative direction, suggesting that Blacks are evaluated more negatively after positive conditioning. Thus, the authors do not show a reliable reduction of negative implicit and explicit prejudice toward Blacks via EC.

In sum, the existing EC research yields initial support for generalization effects in EC but the literature on this effect is still relatively limited. With the present research, we aim to validate the already existing evidence and to contribute to a more consistent picture regarding generalization effects in EC. In particular, we investigate whether evaluatively conditioned attitudes generalize (a) to stimuli that belong to the same category as the CS and (b) to the category itself. In addition, we examine the role of CA in order to learn more about the potential processes underlying generalization effects in EC.

## Overview of Present Research

Four experiments were designed to investigate generalization effects within a standard EC picture-picture paradigm. Positive and negative pictures (USs) were either paired with pictures of members of fictitious alien tribes (CSs in Experiments 1 and 2) or with pictures of employees of two different companies (schematic CSs in Experiment 3 and naturalistic CSs in Experiment 4). Subsequent to the conditioning procedure, evaluative ratings of the CSs, novel category exemplars, and the category itself were assessed. We hypothesized that evaluatively conditioned attitudes would generalize to novel members of the respective CS category as well as to the category itself. CA was assessed (Experiments 1 and 4) or directly influenced via an attention manipulation (Experiments 2 and 3) as a potential moderator of the generalization effects.

### Ethics Statement

In all studies, all participants provided oral informed consent. We did not obtain written informed consent in order to protect participants’ anonymity. The experimenter documented consent by making a note in the research protocol. This consent procedure as well as the procedure of all experiments was approved by the Ethics Committee of the University of Bielefeld. Labeled datasets from the studies may be obtained by writing to the first author.

## Experiment 1

**FIGURE 1:**
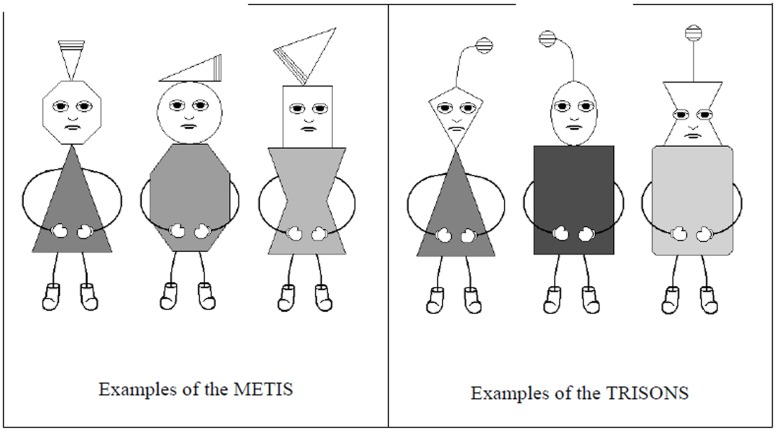
**Examples of the CSs (METIS and TRISONS) used in Experiment 1**.

In Experiment 1, we examined whether generalization effects can occur in EC and whether these effects depend on the salience of the category of the CSs. This idea is based on findings of transfer effects in intergroup contact research (see Brown and Hewstone, 2005), which indicate that some degree of category salience is a necessary precondition for generalization effects in prejudice reduction to occur, whereby it is unclear whether categories need to be salient *during* intergroup contact or whether the category can also be introduced *after* the contact situation. Likewise, generalization effects within EC might also depend on the salience of the CS category during the acquisition phase. In the studies by Spruyt et al. (2014), the attention allocation manipulation was realized either before or during the conditioning procedure. However, it is unclear whether generalization can take place when the CS categories are only established *after* conditioning. We therefore manipulated category salience during conditioning by varying the point in time when participants learned about the category membership of the CSs (*information before EC* condition vs. *information after EC* condition). We expected stronger generalization effects for new exemplars of the CS category as well as for the category itself in the *information before EC* as compared to the *information after EC* condition. The CSs that were used in Experiment 1 were pictures of “alien creatures” of two different “tribes” (METIS = M and TRISONS = T, see **Figure 1**).

### Participants and Design

Participants were *N* = 124 students (54 male, 70 female). Data of 13 participants were excluded from the analyses because they had already participated in a similar experiment or were aware of the purpose of the study. Accordingly, 111 participants (47 male, 64 female) remained in the data set. Participants were randomly assigned to one of four conditions resulting from a 2 (category information: before EC vs. after EC) by 2 (valence: M positive T negative vs. M negative T positive) between-subjects design.

### Materials and Procedure

Participants were seated in front of a computer screen and asked to imagine they were scientists supposed to do research on an alien planet named Elpo and therefore get to know the aliens living there (see Glaser and Walther, 2012). The USs were taken from the International Affective Picture System (IAPS; Lang et al., 2005) and the CSs were pictures of drawings of “alien creatures.” We used artificial creatures to prevent a potential influence of prior knowledge about the groups and to avoid ethical problems with conditioning real groups negatively. Importantly, half of the participants learned about the group membership of the aliens directly *before* EC, whereas the other half learned about the groups and the specific group features directly *after* EC. Specifically, participants were told that two tribes were living on the planet Elpo: the TRISONS (T) and the METIS (M). Participants could distinguish the tribes by their specific headdress: TRISONS had an antenna on their head while METIS had a triangle on their head (see **Figure 1**). The individual TRISONS and METIS differed from each other by the exact look of the headdress, the shape of their head and the shades of gray of their clothing.

In the conditioning phase, participants were told that the aliens would be presented along with landscape pictures (IAPS, Lang et al., 2005) supposedly taken in the areas where the aliens live (USs). In one experimental condition, each of four CS METIS was paired with a negative US and each of four CS TRISONS was paired with a positive US (M-T+). In the other condition, this assignment was reversed (M+T-). Each CS–US pair was presented six times in a trace conditioning procedure, resulting in 48 trials. The CS was presented in the center of the screen for 1500 ms, followed by the US for 1500 ms. The inter-stimulus interval (ISI) was 100 ms and the inter-trial interval (ITI) was 1500 ms. The CS–US pairs were fixed randomized, that is, for each participant a particular CS was always paired with the same US. The order of CS–US pairs was randomized for each participant.

In the test phase, the valence ratings of the eight CSs and of eight generalization stimuli (GSs, i.e., members of the two tribes that were not presented during conditioning) were assessed. All stimuli were presented in randomized order. Participants had to indicate how much they like each of the presented stimuli. Each stimulus was presented in the middle of the screen with a graphic rating scale below which was labeled “don’t like at all” on the left and “like very much” on the right. The graphic scale consisted of no additional numbers or labels. The computer program recorded dislikable judgments on the left side from -100 to -1, and likable judgments on the right side from +1 to +100. The neutral midpoint of the scale (0) served as the starting position for each judgment. In addition, participants were asked how much they like the METIS and TRISONS. They evaluated the tribes using the same graphic rating scale. Finally, participants had to indicate which tribe they would rather seek contact with during a stay on Elpo (-100 = METIS; +100 = TRISONS). The values for this measure were recoded such that positive values indicate a preference for METIS over TRISONS.

Finally, CA was assessed by measuring participants’ awareness of the valence of the pairings (Stahl et al., 2009). Each CS was presented once during the awareness test. Participants had to decide whether the US picture that was presented directly after each alien was a positive or negative picture. To answer, participants had to press keys that were marked with a P (for positive) or an N (for negative). A correct answer was coded 1 and an incorrect answer was coded 0. The mean of all eight trials was used as a CA index, with higher values indicating higher levels of CA. A mean value of 0.50 reflects the chance level of CA. In a final questionnaire, demand awareness was assessed asking participants about their guess regarding the purpose of the study.

### Results

#### Test of EC and Generalization Effects

**FIGURE 2:**
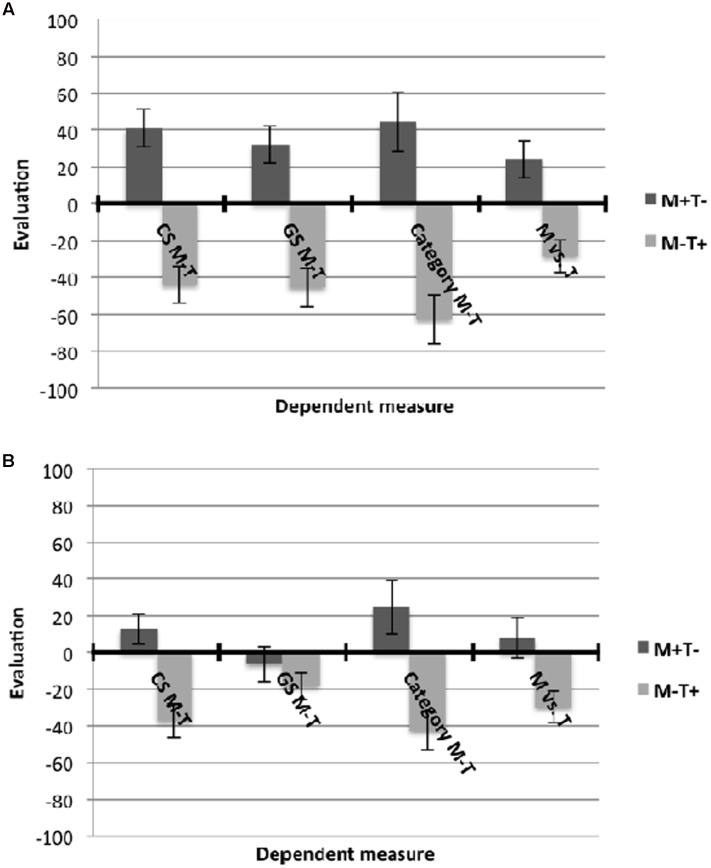
**Means and standard errors of all dependent variables in Experiment 1 in the** **(A)** information before EC condition, and **(B)** information after EC condition. Positive values indicate a preference for METIS over TRISONS while negative values indicate a preference for TRISONS over METIS.

Evaluative conditioning and generalization effects were analyzed using difference scores (M–T) as dependent variables in all analyses. Positive scores indicate a preference of METIS over TRISONS. All means are displayed in **Figures 2A,B**.

##### EC effect

Results of an ANOVA with category information condition and valence condition as between-subjects factors revealed a main effect of valence condition, *F*(1, 107) = 50.69, *p* < 0.001, $\eta_{p}^{2}$ = 0.32. As expected, the CS M–T score differed significantly between the valence conditions (*M* = -40.67, *SD* = 55.67 for M-T+ vs. *M* = 33.28, *SD* = 51.88 for M+T-). No effect of category information condition could be observed, *F*(1, 107) = 1.09, *p* = 0.30, $\eta_{p}^{2}$ = 0.01, and no interaction effect, *F*(1, 107) = 2.73, *p* = 0.10, $\eta_{p}^{2}$ = 0.03.

##### Generalization effect for GSs

Results showed a main effect of valence condition, *F*(1, 107) = 23.15, *p* < 0.001, **$\eta_{p}^{2}$** = 0.18, which was qualified by a valence by category information interaction effect, *F*(1, 107) = 8.59, *p* = 0.004, $\eta_{p}^{2}$ = 0.07. In the *information before EC* condition, GS M–T significantly differed between the valence conditions in the hypothesized direction (*M* = -45.28, *SD* = 59.42 for M-T+ vs. *M* = 33.87, *SD* = 59.96 for M+T-), *t*(55) = 4.97, *p* < 0.001, *d* = 1.34 (**Figure 2A**). In contrast, in the *information after EC* condition, this difference failed to reach significance (*M* = -18.71, *SD* = 41.17 for M-T+ vs. *M* = 0.51, *SD* = 51.20 for M+T-), *t*(52) = 1.53, *p* = 0.13, *d* = 0.42. However, a correlation of the EC score and the GS score in the *information after EC* condition revealed substantial correlations, Cronbachs α = 0.48, *p* < 0.001, indicating that there is meaningful variance in the generalization effect in the *information after EC* condition.

**FIGURE 3:**
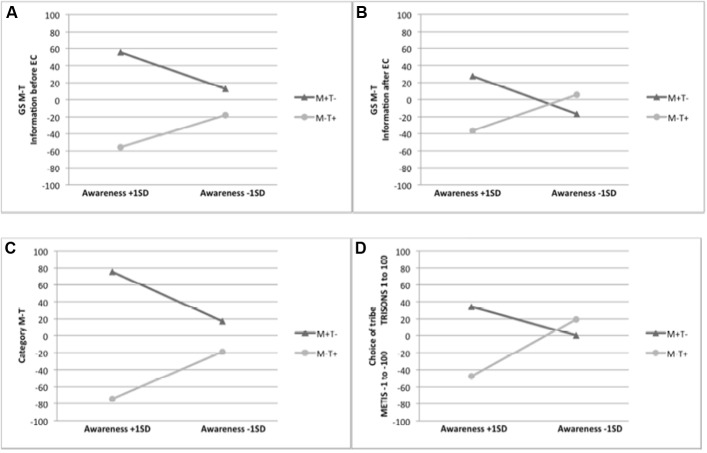
**Conditional generalization effects of evaluative conditioning in Experiment 1 at 1 SD above and below the mean of participants’ CA on** **(A)** the generalization stimuli difference scores (GS M–T) in the information before EC condition, **(B)** the generalization stimuli difference scores (GS M–T) in the information after EC condition, **(C)** the category evaluation difference score (category M–T) across both information conditions, and **(D)** the choice of tribe across both information conditions.

To investigate whether the reported generalization effects would be moderated by CA^1^, we conducted moderated regression analyses using the SPSS Macro PROCESS (Hayes, 2013)^2^. In the *information before EC* condition, the analysis revealed that the interaction term had a significant effect [*b* = 228.24, *SE* = 70.42, Δ*R*^2^ = 0.11, *F*(1, 53) = 10.50, *p* = 0.002], resulting in a significant overall model with *R*^2^ = 0.43, *F*(3, 53) = 13.50, *p* < 0.001. This demonstrates that the generalization effect on GS M–T is moderated by participants’ CA. The simple slope analysis revealed a generalization effect for GS M–T given high CA (*b* = 111.48, *SE* = 17.52, *p* < 0.001) but not given low CA (*b* = 30.99, *SE* = 21.72, *p* = 0.16; **Figure 3A**). In the *information after EC* condition, the interaction term was significant [*b* = 181.78, *SE* = 48.41, Δ*R*^2^ = 0.21, *F*(1, 50) = 14.10, *p* < 0.001], resulting in a significant overall model with *R*^2^ = 0.25, *F*(3, 58) = 6.51, *p* < 0.001. The simple slope analysis revealed a generalization effect for GS M-T given high CA (*b* = 63.93, *SE* = 16.56, *p <* 0.001) but no generalization given low CA (*b* = -22.60, *SE* = 16.06, *p* = 0.17; **Figure 3B**).

##### Generalization effect for category evaluation

A main effect of valence condition emerged, *F*(1, 107) = 42.58, *p* < 0.001, $\eta_{p}^{2}$ = 0.29, demonstrating that the category M–T score is significantly different between the valence conditions (*M* = -53.57, *SD* = 68.40 for M-T+ vs. *M* = 42.19, *SD* = 84.90 for M+T-). The moderated regression analyses for the difference score category M–T revealed a significant interaction effect [*b* = 258.56, *SE* = 57.43, Δ*R*^2^ = 0.11, *F*(1, 107) = 20.27, *p* < 0.001], resulting in a significant overall model, *R*^2^ = 0.37, *F*(3, 120) = 23.58, *p* < 0.001. According to the simple slope analysis, a generalization effect on category M–T was observed only when participants scored high on CA (*b* = 149.96, *SE* = 17.99, *p* < 0.001) but not when participants scored low on CA (*b* = 36.07, *SE* = 19.25, *p* = 0.06; **Figure 3C**), although the latter effect approached statistical significance.

##### Generalization effect for choice of tribe

The ANOVA yielded a main effect of valence condition, *F*(1, 107) = 22.45, *p* < 0.001, $\eta_{p}^{2}$ = 0.17. Participants preferred TRISONS over METIS in the M-T+ (*M* = -28.84, *SD* = 48.51) and METIS over TRISONS in the M+T- condition (*M* = 20.25, *SD* = 59.32). Regarding the moderated regression analysis, the interaction term showed a significant effect [*b* = 143.14, *SE* = 41.01, Δ*R*^2^ = 0.08, *F*(1, 107) = 12.18, *p* < 0.001], resulting in a significant overall model, *R*^2^ = 0.29, *F*(3, 107) = 14.31, *p* < 0.001. A generalization effect on choice of tribe emerged when participants scored high on CA (*b* = 81.98, *SE* = 12.85, *p* < 0.001) but not when participants scored low on CA (*b* = 19.08, *SE* = 13.75, *p* = 0.17; **Figure 3D**).

### Discussion

Experiment 1 revealed that EC effects generalize to other members of the CS category as well as to the category itself. Importantly, salience of the CS category as well as CA played an important role for EC generalization effects. For a clearer understanding of the findings, we need to distinguish between evaluations on the category level and the exemplar level.

On the category level, generalization effects in both information conditions were moderated by CA, with significant effects for participants high but no effects for participants low in CA. Although effects on the category level were obtained in both information conditions, they might be explained with different processes. In the *information before EC* condition, not only the exemplars of the categories but also the categories themselves might have served as CSs during the conditioning procedure. Consequently, the generalization effect on the category level might partly represent an EC effect. In contrast, in the *information after EC* condition, only the exemplars (and not the category) could serve as CSs because the category was unknown for participants during conditioning. Thus, when the category information was given *after* the EC procedure, the effect on the category level represents a generalization effect. In this case, generalization might be due to inferences participants made about the category after the EC procedure. The finding that category generalization effects were obtained only when CA was high is consistent with this explanation because such inferences can be made only when participants can recall what happened during the EC procedure. This reasoning is in line with research by Gast and De Houwer (2012) showing that EC can also be obtained when knowledge about the stimulus relation is inferred rather than experienced directly. Taken together, on the category level, salience of the category during the conditioning procedure is not necessary for obtaining generalization effects; rather, the category information can be integrated afterward. One limitation that has to be mentioned is that we cannot rule out the possibility that at least some of the participants in the *information after EC* condition noticed the difference between the categories spontaneously during conditioning. However, this seems unlikely because the stimuli differed on a few attributes and not just on the actual group-defining feature. Moreover, participants had no reason to assume that the presented stimuli belong to different categories because there were also many similarities between the stimuli.

On the exemplar level, generalization effects were initially obtained in the *information before EC* condition but not in the *information after EC* condition, indicating that—on the exemplar level—category salience is an important precondition. Although this seems to be in line with intergroup contact research (Brown and Hewstone, 2005), the pattern of results changes when taking participants’ CA into account. Specifically, in both information conditions, a generalization effect on the exemplar level was found when participants were high in CA but not when they were low in CA. Consequently, as long as CA is high a generalization effect on the exemplar level can be obtained, irrespective of category salience.

## Experiment 2

In Experiment 2, we investigated the influence of CA on EC generalization more directly by manipulating the focus of attention which is supposed to influence CA. We hypothesized that generalization effects would be most pronounced in the condition with high CA and smallest in the condition with low CA, with the control condition in between.

### Participants and Design

*N* = 153 students (74 male, 79 female) participated in the experiment. Data from nine participants were excluded, either because of technical problems (*n* = 3) or because participants were aware of the purpose of the study (*n* = 6). Thus, *N* = 144 participants (68 male, 76 female) remained in the data set. Participants were randomly assigned to one of six conditions resulting from a 2 (valence: M+T- vs. M-T+) × 3 (attention manipulation: control vs. distraction vs. focus) between-subjects design.

### Materials and Procedure

The same material and cover story as in Experiment 1 was used. However, in Experiment 2, only the *information before EC* condition was implemented. Furthermore, an attention manipulation was implemented in the conditioning phase. Participants in the control condition were simply asked to watch the presentation of the pictures. In the focus condition, participants were instructed to watch the presentation of the pictures and to remember the CS–US pairings. In the distraction condition, a secondary task was introduced in order to decrease CA (cf. Kattner, 2012). Specifically, the letters “X” and “O” were displayed in varying corners of the screen. While watching the presentation of the pictures, participants also had to attend to the letters and were asked to press the key marked with “X” or the key marked with “O” depending on which letter was displayed. Furthermore, participants in the distraction condition were instructed to remember how many “X” and “O” would be displayed. In this condition, the CS always appeared for 1000 ms alone and was then joined by an “X” or “O” that was displayed together with the CS for 500ms, irrespective of the participants’ reaction times. After the test phase, participants in the distraction condition had to indicate how many “X” and “O” they saw during conditioning.

Subsequently, we checked whether our attention manipulation successfully influenced CA using a slightly different procedure for assessing CA as in the previous study. Specifically, participants were presented each CS along with all USs that had been used in the study and had to indicate which US had been paired with the respective CS. We examined whether participants picked a US of the correct valence. A correct answer (i.e., correct valence of the US) was coded 1 and an incorrect answer was coded 0. The mean of all eight trials was used as a CA index, with higher values indicating higher levels of CA. A mean value of 0.50 reflects the chance level of CA.

### Results

#### Manipulation Check

We investigated whether the attention manipulation affected participants’ CA. Results of a 2 (valence: M+T- vs. M-T+) × 3 (attention manipulation: control vs. distraction vs. focus) between-subjects ANOVA with the mean CA score as the dependent variable revealed a main effect of attention condition, *F*(2, 137) = 52.97, *p* < 0.001, $\eta_{p}^{2}$ = 0.44, but no main effect of valence condition, *F*(1, 137) = 1.28, *p* = 0.26, $\eta_{p}^{2}$ = 0.009, and no interaction effect, *F*(2, 137) = 1.20, *p* = 0.30, $\eta_{p}^{2}$ = 0.017. Participants in the distraction condition showed lower levels of CA (*M* = 0.49, *SD* = 0.25) than did participants in the focus condition (*M* = 0.91, *SD* = 0.19), *t*(92) = 9.25, *p* < 0.001, *d* = 1.94, and in the control condition (*M* = 0.88, *SD* = 0.22), *t*(95) = 8.16, *p* < 0.001, *d* = 1.67. The focus and the control condition did not differ from each other, *t*(93) = 0.72, *p* = 0.48.

#### Test of EC and Generalization Effects

As in Experiment 1, EC and generalization effects were analyzed using difference scores (M–T) as dependent variables. Positive scores indicate a preference for METIS over TRISONS.

##### EC effect

**FIGURE 4:**
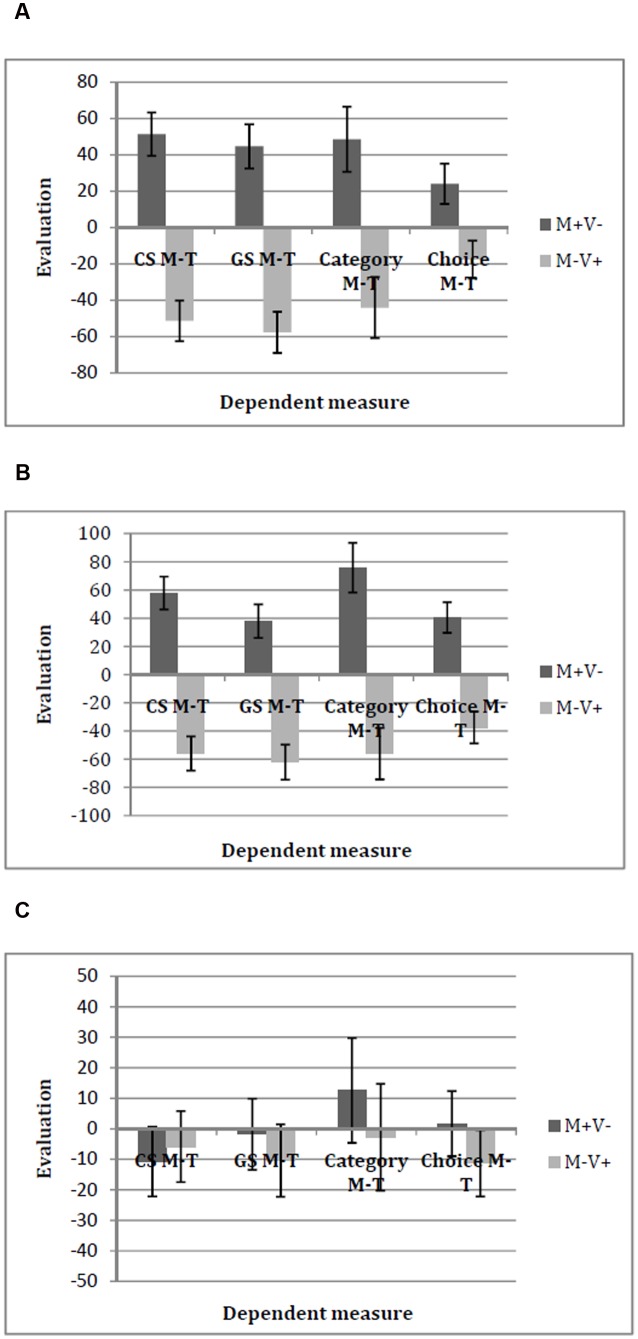
**Means and standard errors of all dependent variables in Experiment 2 in** **(A)** the focus condition, **(B)** the control condition, and **(C)** the distraction condition. Positive values indicate a preference for METIS over TRISONS while negative values indicate a preference for TRISONS over METIS.

Results of a 2 (valence: M+T- vs. M-T+) × 3 (attention manipulation: control vs. distraction vs. focus) between-subjects ANOVA with the CS M–T score as the dependent variable revealed a main effect of valence condition, *F*(1, 138) = 55.15, *p* < 0.001, $\eta_{p}^{2}$ = 0.29, which was qualified by a significant interaction effect of valence by attention manipulation, *F*(2, 138) = 16.02, *p* < 0.001, $\eta_{p}^{2}$ = 0.19. *Post hoc* tests showed an EC effect in the focus condition (*M* = 57.91, *SD* = 53.56 for M+T- vs. *M* = -55.95, *SD* = 81.4 for M-T+), *t*(44) = 5.65, *p* < .001, *d* = 1.69 (**Figure 4A**), and in the control condition, (*M* = 51.42, *SD* = 58.49 for M+T- vs. *M* = -51.26, *SD* = 61.97 for M-T+), *t*(47) = 5.94, *p* < 0.001, *d* = 1.72 (**Figure 4B**). In the distraction condition, no EC effect was found, (*M* = -10.76, *SD* = 45.66 for M+T- vs. *M* = -5.86, *SD* = 29.93 for M-T+), *t*(47) = 0.44, *p* = 0.66 (**Figure 4C**).

##### Generalization effect for GSs

The ANOVA with the GS M–T score as the dependent variable yielded a main effect of valence condition, *F*(1, 138) = 52.52, *p* < 0.001, $\eta_{p}^{2}$ = 0.28, and a significant interaction effect, *F*(2, 138) = 10.24, *p* < 0.001, $\eta_{p}^{2}$ = 0.13. As **Figure 4** shows, we found a generalization effect on the GSs in the focus condition (*M* = 37.97, *SD* = 59.59 for M+T- vs. *M* = -62.02, *SD* = 80.6 for M-T+), *t*(44) = 4.81, *p* < 0.001, *d* = 1.44, and in the control condition (*M* = 44.68, *SD* = 64.42 for M+T- vs. *M* = -57.63, *SD* = 66.41 for M-T+), *t*(47) = 5.46, *p* < 0.001, *d* = 1.58. For the distraction condition, no such generalization effect on the GSs was found, *t*(47) = 0.96, *p* = 0.34. Thus, the generalization effects mirror the obtained EC effects.

##### Generalization effect for category evaluation

Results for the category M–T score revealed a main effect of valence condition, *F*(1, 138) = 31.1, *p* < 0.001, $\eta_{p}^{2}$ = 0.18, and a significant interaction of valence by attention manipulation, *F*(2, 138) = 5.7, *p* = 0.004, $\eta_{p}^{2}$ = 0.08. In the focus condition, participants preferred METIS over TRISONS in the M+T- condition (*M* = 75.83, *SD* = 66.93), whereas they showed a preference for TRISONS over METIS in the M-T+ condition, (*M* = -56, *SD* = 113.23), *t*(44) = 4.86, *p* < 0.001, *d* = 1.45. This effect was also significant in the control condition (*M* = 48.61, *SD* = 85.36 for M+T- vs. *M* = -44.04, *SD* = 101.55 for M-T+), *t*(47) = 3.43, *p* = 0.001, *d* = 0.99. In the distraction condition, no generalization effect could be observed, *t*(47) = 0.77, *p* = 0.45.

##### Generalization effect for choice of tribe

The ANOVA yielded a main effect of valence condition, *F*(1, 138) = 24.72, *p* < 0.001, $\eta_{p}^{2}$ = 0.15, as well as an interaction effect, *F*(2, 138) = 4.42, *p* = 0.014, $\eta_{p}^{2}$ = 0.06. *Post hoc* tests showed a generalization effect in the focus condition (*M* = 40.54, *SD* = 42.65 for M+T- vs. *M* = -37.45, *SD* = 60.6 for M-T+), *t*(44) = 5.08, *p* < 0.001, *d* = 1.52 (**Figure 4A**), and in the control condition, (*M* = 24.13, *SD* = 60.92 for M+T- vs. *M* = -17.54, *SD* = 58.62 for M-T+), *t*(47) = 2.44, *p* = 0.019, *d* = 0.71 (**Figure 4B**). In the distraction condition, no generalization effect was found, *t*(47) = 0.96, *p* = 0.34 (**Figure 4C**).

### Discussion

Experiment 2 nicely replicates the findings obtained in Experiment 1. Moreover, Experiment 2 yields evidence that CA has a causal moderating influence on EC and on generalization effects. EC and generalization effects (on all dependent variables) were reliably obtained in the focus and control condition where actual contingency awareness was quite high. In contrast, when CA was reduced by distracting participants during the conditioning procedure, there were no significant EC and as a consequence also no generalization effects at all. Interestingly, the effects were obtained on all dependent variables, indicating that generalization worked equally well on the category and on the exemplar level, at least when CA is high. It seems that EC effects in our paradigm are based on propositional processes as compared to associative processes because merely associative EC effects do not require CA. However, one could argue that the distraction (i.e., reacting to *and* counting the letters) was so strong that relatively automatic associative processes were also disabled, resulting in no EC (and no generalization) in the distraction condition. In other words, the conditions that interfere with propositional reasoning may also interfere with associative processing (see e.g., Gilbert and Hixon, 1991). We address this issue in Experiment 3 by adjusting the distraction manipulation.

## Experiment 3

The aim of Experiment 3 was to replicate our previous findings with different stimulus materials in order to rule out that the effects are restricted to the specific materials or cover story that we used. In Experiment 3, we used drawings of humans as CSs that were divided into two fictitious groups, allegedly being employees of two different companies. In a pilot study, *N* = 60 participants evaluated 23 fictitious names of companies according to their likeability. For Experiment 3, two neutral company names were selected: AERU (A) and VOLLO (V).

### Participants and Design

*N* = 123 students (53 male, 70 female) participated in the experiment. Data from five participants were excluded, as they had already participated in one of the experiments reported above or were aware of the purpose of the study. Thus, *N* = 118 participants (49 male, 69 female) remained in the data set. Participants were randomly assigned to one of six conditions resulting from a 2 (valence: A+V- vs. A-V+) × 3 (attention manipulation: control vs. distraction vs. focus) between-subjects design.

### Materials and Procedure

**FIGURE 5:**
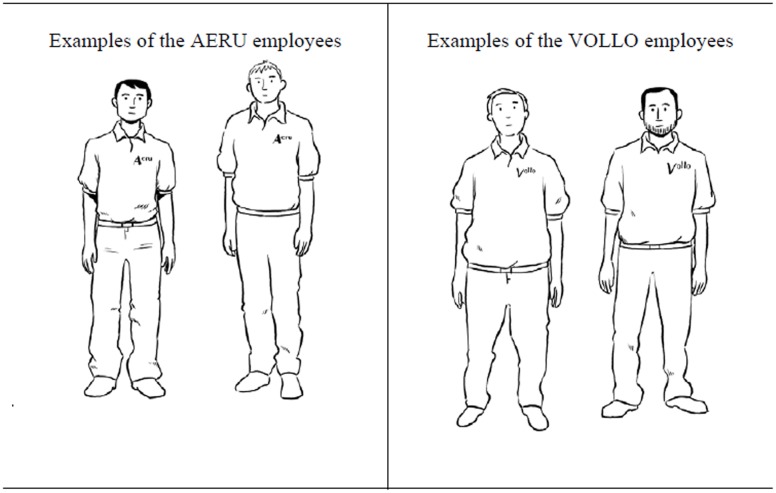
**Examples of the CSs (employees of the AERU and VOLLO companies) used in Experiment 2.** The company name is always displayed on the shirt of each exemplar.

Participants were seated in front of a computer screen and were instructed to imagine that they were a member of the city council and had to decide which of two electronics companies (AERU and VOLLO) should be allowed to open a store in their city. They were told that they would first be introduced to employees of both companies. Therefore, photographs of the employees (CSs) would be presented along with pictures of the employee’s last vacation (USs). The CSs were drawn pictures of male individuals. Participants were informed that the name of the company that the persons work for would be printed on the shirts of the employees (**Figure 5**). The positive USs were pictures of a beach, a waterfall, an ocean view, and a mountain panorama. The negative USs were pictures of cigarette butts, a wooden floor with chunks of dust, a beach filled with garbage, and a moldy room corner.

In one experimental condition (V+A-), each of four employees of the VOLLO company was paired with a positive US and each of four employees of the AERU company was paired with a negative US. In the other condition (V-A+), the valence assignment was reversed. The attention manipulation was the same as in Experiment 2 with the exception that participants in the distraction condition only had to react to the “X” and “O” and did not have to count the letters. In all three awareness conditions, each CS–US pair was presented eight times in a trace conditioning procedure, resulting in 64 trials. In the test phase, the same graphic rating scale as in the previous studies was used. The valence ratings of the CSs were assessed first. Subsequently, participants were asked to evaluate other employees of the AERU company and then of the VOLLO company (GSs). All GSs were presented without the company logo on their shirts in order to ensure that the generalization effect expected for the GSs is not caused by the conditioning of the respective company logo. However, participants were informed prior to giving their evaluations that the persons that would now be presented work for either AERU or VOLLO, respectively. Subsequently, participants rated how much they liked the two companies using the same rating scale, which was now labeled “don’t like at all” (-100) on the left and “like very much” (+100) on the right. Finally, as a manipulation check, participants also rated the valence of the USs on a scale from -100 (negative) to +100 (positive). In the last part of the experiment, we checked whether our attention manipulation was successful using the same procedure for assessing CA as in Experiment 1.

### Results

#### Preliminary Analyses

##### Manipulation check I

We first checked whether the USs were indeed perceived as positive and negative, respectively, by testing the US ratings against zero. Results confirmed that the positive USs were evaluated positively, *t*(117) = 45.12, *p* < 0.001, *d* = 4.15 (*M* = 78.77, *SD* = 18.96), while the negative USs were evaluated negatively, *t*(117) = -52.29, *p* < 0.001, *d* = 4.81 (*M* = -87.75, *SD* = 18.23).

##### Manipulation check II

With three *t*-tests, we investigated whether the attention manipulation affected participants’ CA. Participants in the distraction condition showed lower levels of CA (*M* = 0.73, *SD* = 0.28) than did participants in the focus condition (*M* = 0.95, *SD* = 0.12), *t*(79) = 4.63, *p* < 0.001, *d* = 1.04, and in the control condition (*M* = 0.91, *SD* = 0.20), *t*(76) = 3.25, *p* = 0.002, *d* = 0.75. The focus and the control condition did not differ from each other, *t*(75) = 1.10, *p* = 0.27, *d* = 0.25.

#### Test of EC and Generalization Effects

As in the previous experiments, EC and generalization effects were analyzed using difference scores (A-V) as dependent variables. Positive scores indicate a preference for AERU over VOLLO.

##### EC effect

**FIGURE 6:**
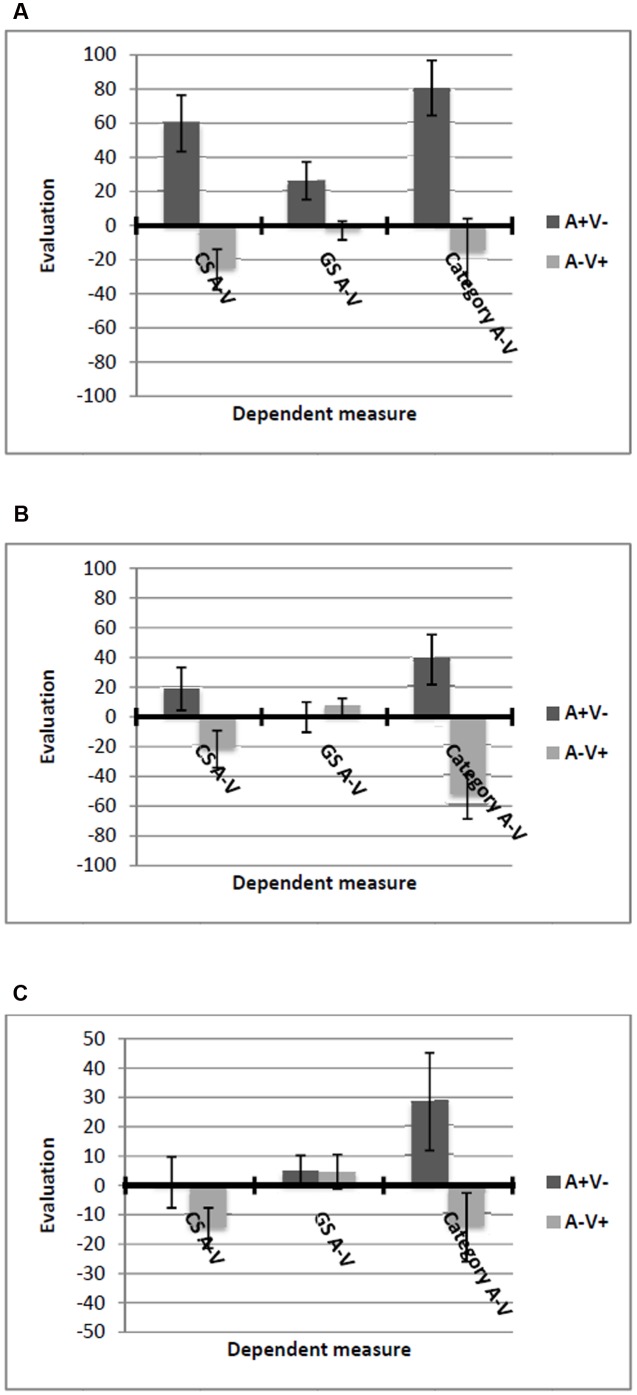
**Means and standard errors of all dependent variables in Experiment 3 in** **(A)** the focus condition, **(B)** the control condition, and **(C)** the distraction condition. Positive values indicate a preference for AERU over VOLLO while negative values indicate a preference for VOLLO over AERU.

Results of a 2 (valence: A+V- vs. A-V+) × 3 (attention manipulation: control vs. distraction vs. focus) between-subjects ANOVA with the CS A–V score as the dependent variable revealed a main effect of valence condition, *F*(1, 112) = 22.63, *p* < 0.001, $\eta_{p}^{2}$ = 0.17, which was qualified by a significant interaction effect of valence by attention manipulation, *F*(2, 112) = 4.39, *p* = 0.02, $\eta_{p}^{2}$ = 0.07. *Post hoc* tests showed an EC effect in the focus condition (*M* = 59.86, *SD* = 73.84 for A+V- vs. *M* = -25.79, *SD* = 53.04 for A-V+), *t*(38) = 4.21, *p* < 0.001, *d* = 1.37 (**Figure 6A**), and in the control condition, (*M* = 18.82, *SD* = 62.64 for A+V- vs. *M* = -21.69, *SD* = 52.70 for A-V+), *t*(35) = 2.12, *p* = 0.04, *d* = 0.72 (**Figure 6B**). In the distraction condition, no significant EC effect was found, (*M* = 1.04, *SD* = 39.92 for A+V- vs. *M* = -14.48, *SD* = 30.86 for A-V+), *t*(39) = 1.39, *p* = 0.17, *d* = 0.45 (**Figure 6C**).

##### Generalization effect for GSs

The ANOVA with the GS A–V score as the dependent variable yielded no main effects [main effect valence: *F*(1, 112) = 1.47, *p* = 0.28, $\eta_{p}^{2}$ = 0.01; main effect attention: *F*(1, 112) = 0.71, *p* = 0.5, $\eta_{p}^{2}$ = 0.01] but a significant interaction effect, *F*(2, 112) = 3.08, *p* = 0.05, $\eta_{p}^{2}$ = 0.05. As **Figure 6A** shows, we found a generalization effect on the GSs in the focus condition (*M* = 26.25, *SD* = 49.19 for A+V- vs. *M* = -2.93, *SD* = 24.49 for A-V+), *t*(38) = 2.37, *p* = 0.02, *d* = 0.77. For the control condition and the distraction condition, no such generalization effects on the GSs were found [control: *t*(35) = 0.55, *p* = 0.58; distraction: *t*(39) = 0.021, *p* = 0.86].

##### Generalization effect for category evaluation

Results for the category A–V score revealed a main effect of valence condition, *F*(1, 112) = 34.00, *p* < 0.001, $\eta_{p}^{2}$ = 0.23. Participants preferred AERU over VOLLO in the A+V- condition (*M* = 49.10, *SD* = 76.28), whereas they showed a preference for VOLLO over AERU in the A-V+ condition, (*M* = -26.76, *SD* = 70.06). Unexpectedly, we also found a main effect of attention manipulation, *F*(2, 112) = 3.13, *p* = 0.05, $\eta_{p}^{2}$ = 0.05. Participants in the focus condition showed a general preference for AERU over VOLLO (*M* = 32.73, *SD* = 92.05) compared to the other two attention conditions (*M* = 7.68, *SD* = 68.47 for distraction; *M* = -6.22, *SD* = 82.43 for control). However, this main effect has no implications for our hypotheses. Importantly, no interaction effect could be observed, *F*(2, 112) = 1.72, *p* = 0.18, $\eta_{p}^{2}$ = 0.03, indicating that generalization on the category didn’t depend on awareness.

### Discussion

Experiment 3 revealed that generalization effects within an EC paradigm can be obtained when using different and socially more relevant stimuli. Most importantly, Experiment 3 confirmed that CA has a causal moderating influence on EC and on generalization effects. When CA was enhanced by instructing participants to encode the CS–US pairings, EC as well as generalization effects (on all dependent variables) were observed. In contrast, when CA was reduced by distracting participants during the conditioning procedure, there were no significant EC and generalization effects on the exemplar level. On the category level, however, the level of awareness did not influence generalization (see General Discussion for possible explanations).

One unexpected finding was obtained in the control condition. Although there was a significant EC effect in the control condition, no generalization on the GSs could be observed. This might be due to the relatively small EC effect in the control condition as compared to the focus condition of this experiment and to the control condition in Experiment 2. The small EC effect may have made generalization (without focus on the contingencies) unlikely.

## Experiment 4

**FIGURE 7:**
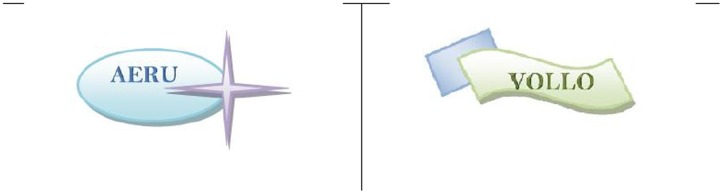
**Logos used in Experiment 4**.

Although these experiments provide consistent evidence for generalization effects, one could still critically argue that all experiments used schematic stimuli and that generalization might work differently when using naturalistic and thus more complex stimuli (cf. Hütter et al., 2013). Specifically, schematic stimuli are always impoverished in terms of the variability of individuating features, increasing the likelihood that only the category itself is used as the judgmental basis. Naturalistic stimuli, in contrast, are heterogeneous and provide many individual characteristics that contribute to an evaluation, thereby making it potentially more difficult to obtain generalization effects along one specific dimension (i.e., CS category). The aim of Experiment 4 was to investigate whether generalization effects within an EC paradigm can also be obtained when using naturalistic, socially relevant stimuli. We used pictures of real persons as CSs in this study. The cover story was the same as in Experiment 3. In Experiment 4, the group-defining feature was the name of the company (AERU or VOLLO) written within a logo (**Figure 7**). Two neutral logos were selected based on the results of a pilot study in which *N* = 60 participants evaluated 12 fictitious logos according to their likeability. In Experiment 4, the respective logo and company name was placed in the upper right corner of the CS pictures.

Additionally, we assessed evaluations of the two company logos in order to test whether the logos themselves served as the effective CSs and thus acquired the conditioned valence. Furthermore, in Experiment 3, we obtained generalization effects although the company name was not present when evaluating the GSs. However, it is plausible that generalization effects might be stronger when the GSs are presented together with the group-defining feature. In Experiment 4, we therefore varied whether the GSs were presented with or without the logo in the test phase.

### Participants and Design

Participants were *N* = 127 students (59 male, 68 female). Data from nine participants were excluded, as they were aware of the hypotheses or participated in one of the earlier experiments reported above. Thus, 118 participants (55 male, 63 female) remained in the data set. Participants were randomly assigned to a 2 (valence: A+V- vs. A-V+) × 2 (logo presentation of the GSs in the test phase: with logo vs. without logo) between-subjects design.

### Materials and Procedure

The procedure of Experiment 4 was almost identical to Experiment 3 with the following exceptions: First, the CSs were pictures of male individuals taken from a face database (Minear and Park, 2004). Second, participants were informed that both companies have a logo that would appear on the photographs of the employees. Participants were shown both logos immediately before the conditioning started. Third, in the test phase, the respective company logo was placed on the pictures of the GSs (just as on the CS pictures during conditioning) in one experimental condition; there was no logo present in the other experimental condition. However, all participants were informed prior to giving their ratings whether the presented employees worked for the AERU or VOLLO company, respectively. Thus, in the condition without logo on the GSs, the expected generalization cannot be based on perceptual features but solely on inferences drawn by participants based on presented information. Fourth, evaluations of the two logos were assessed using the same rating scale as before, which was now labeled “don’t like at all” on the left and “like very much” on the right. Finally, CA was measured at the end of the experiment.

### Results

#### Preliminary Analyses

##### Manipulation check

Results of two *t*-tests (against the test value of 0) confirmed that the positive USs were evaluated positively (*M* = 70.94, *SD* = 27.46), *t*(117) = 28.06, *p* < 0.001, *d* = 2.57, while the negative USs were evaluated negatively (*M* = -82.88, *SD* = 29.34), *t*(117) = -30.68, *p* < 0.001, *d* = -2.81.

#### Test of EC and Generalization Effects

**FIGURE 8:**
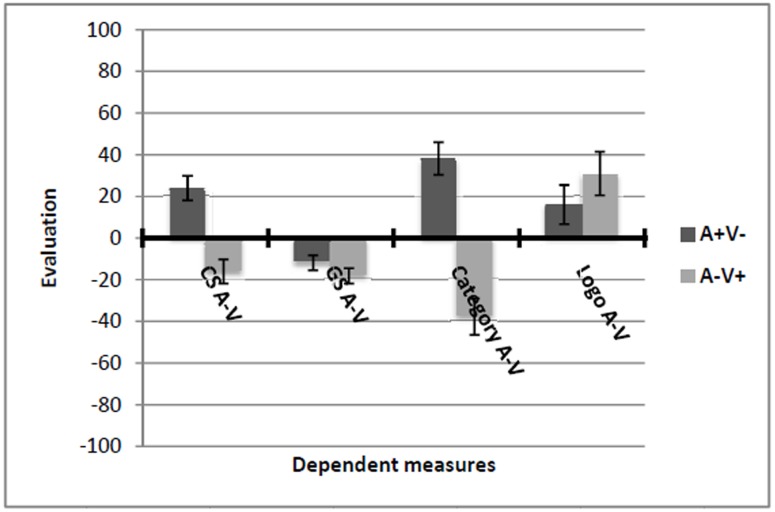
**Means and standard errors of all dependent variables of Experiment 4.** Positive values indicate a preference for AERU over VOLLO while negative values indicate a preference for VOLLO over AERU.

Evaluative conditioning and generalization effects were analyzed using difference scores (A-V) as dependent variables. Positive scores indicate a preference for AERU over VOLLO. All means are displayed in **Figure 8**.

##### EC effect

Results of a *t*-test with valence (A+V- vs. A-V+) as the between-subjects factor and the CS A–V score as the dependent variable revealed that AERU was preferred over VOLLO in the A+V- condition (*M* = 24.03, *SD* = 45.71) while VOLLO was preferred in the A-V+ condition (*M* = -16.10, *SD* = 43.98), *t*(116) = 4.86, *p* < 0.001, *d* = 0.90.

##### Generalization effect for GSs

**FIGURE 9:**
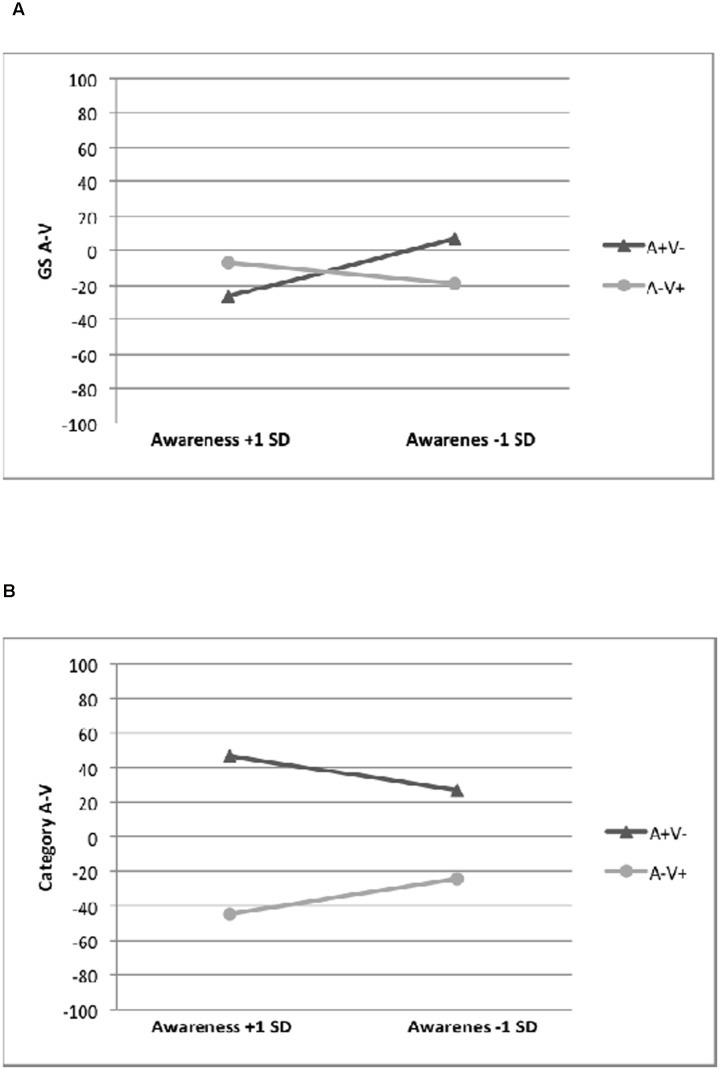
**Conditional generalization effects of evaluative conditioning in Experiment 4 at 1 SD above and below the mean of participants’ CA on** **(A)** the generalization stimuli difference scores (GS A–V) in the logo present condition, and **(B)** the category evaluation difference score (category A–V) across both logo conditions.

For the GS A–V score, the 2 (valence: A+V- vs. A-V+) × 2 (logo on GSs: present vs. not present) between-subjects ANOVA yielded neither a main nor an interaction effect [valence: *F*(1, 118) = 1.43, *p* = 0.23, $\eta_{p}^{2}$ = 0.01; logo: *F*(1, 118) = 1.22, *p* = 0.27, $\eta_{p}^{2}$ = 0.01; interaction: *F*(2, 118) = 1.26, *p* = 0.26, $\eta_{p}^{2}$ = 0.01]. The moderated regression analysis revealed a significant valence condition by CA interaction (*b* = 52.15, *SE* = 19.62, *p* = 0.01) and a three-way interaction of valence condition, logo condition, and CA (*b* = 91.17, *SE* = 39.43, *p* = 0.02). This resulted in a significant overall model, *R*^2^ = 0.17, *F*(7, 108) = 3.07, *p* = 0.01. In order to facilitate interpretation of this result, two regression analyses were run, separately for each logo condition. For the condition in which the GSs were presented without the company logo, the regression analysis did not result in a significant overall model, *R*^2^ = 0.05, *F* < 1. In contrast, in the condition where participants saw the GSs together with the company logo, a significant valence condition by CA interaction emerged [*b* = 94.64, *SE* = 24.24, Δ*R*^2^ = 0.19, *F*(1, 56) = 13.79, *p* < 0.001]. The overall model was significant, *R*^2^ = 0.23, *F*(3, 56) = 5.70, *p* = 0.002. The simple slope analysis revealed a generalization effect for GS A–V given high CA (*b* = 19.73, *SE* = 8.74, *p* = 0.03; **Figure 9A**). There was also a significant effect for participants having low CA (*b* = -25.90, *SE* = 9.94, *p* = 0.01). However, the latter effect was opposite to the expected direction, pointing toward a contrast effect.

##### Generalization effect for category evaluation

As hypothesized, participants preferred AERU over VOLLO in the A+V- condition (*M* = 38.13, *SD* = 60.74) and VOLLO over AERU in the A-V+ condition (*M* = -37.72, *SD* = 66.65), *t*(116) = 6.47, *p* < 0.001, *d* = 1.20. Regarding the moderation, we found a significant interaction effect [*b* = 92.94, *SE* = 45.57, Δ*R*^2^ = 0.03, *F*(1, 112) = 4.16, *p* = 0.04]. The overall regression model was significant, *R*^2^ = 0.29, *F*(3, 112) = 15.06, *p* < 0.001. A generalization effect on category evaluation emerged when participants were high in CA (*b* = 91.48, *SE* = 14.17, *p* < 0.001) and also when participants scored low on CA (*b* = 51.19, *SE* = 16.75, *p* = 0.002; **Figure 9B**).

##### Evaluation of the company logo

Evaluations of the company logos did not differ between experimental conditions, *t*(116) = 1.06, *p* = 0.29, *d* = 0.20. Regarding the moderation with CA, we found no significant interaction effect [*b* = 94.97, *SE* = 62.91, Δ*R*^2^ = 0.02, *F*(1, 112) = 2.28, *p* = 0.13). The overall regression model was also not significant, *R*^2^ = 0.04, *F*(3, 112) = 1.86, *p* = 0.14.

### Discussion

Results of Experiment 4 yielded evidence for generalization effects using naturalistic stimuli. In general, the data pattern looks similar to the previous experiments although some differences emerged as well.

First, generalization on the GSs was found only when the logo was present during the evaluation. This is in contrast to Experiment 3, where effects on the GSs were obtained when they were presented without logo. These diverging results might be due to the complexity of the respective stimulus material. Although the drawn persons in Experiment 3 were clearly distinguishable from each other, they showed less heterogeneity compared to the “real” person stimuli used in Experiment 4. Thus, when using naturalistic instead of impoverished schematic stimuli, the visibility of a group-defining feature might serve as a prerequisite for generalization effects. However, when the group feature was present during the test phase, clear generalization effects were observed on the exemplar level when CA was high, replicating our previous findings. Second, on the category level, we replicated the overall data pattern obtained in the previous experiments: Generalization effects were moderated by CA with more pronounced effects when participants were high in CA.

Interestingly, in the present study, the perceptual group-defining feature (i.e., the logo) was unaffected by the conditioning procedure. With this result, we provide evidence that the individual CS exemplars served as CSs and not just a perceptual feature that all CSs and GSs of one category shared with each other.

## General Discussion

With the present research, we aimed to investigate generalization effects in EC. Specifically, in four experiments, we tested whether attitudes toward specific CSs acquired in an EC procedure would generalize to other exemplars of the CS category as well as to the CS category. In Experiments 1 and 2, schematic drawings of members of two tribes of ‘aliens’ served as CSs, whereas in Experiment 3, the CSs were schematic drawings of human beings. In Experiment 4, photographs of human faces served as CSs. Experiment 1 revealed that EC effects indeed generalized to other exemplars of the CS category (i.e., to other METIS and TRISONS) and to the CS category itself. In Experiment 1, we investigated whether the pre-association between CSs and CS category has to be present during conditioning for generalization effects to occur. We manipulated category salience by providing the information about the CS category either before or after conditioning. This manipulation affected generalization only on the exemplar level (GSs) with generalization in the *information before* but not in the *information after EC* condition. Thus, category salience was indeed a moderator for the generalization effects. Moreover, CA moderated generalization for the GSs in both information conditions with significant generalization for participants high but no effects for participants low in CA. At the category level, generalization was not affected by the category salience manipulation but instead depended solely on CA. In Experiment 2, we manipulated CA (via an attention manipulation) and found EC and generalization effects in the focus and control condition. In the distraction condition, no significant EC and generalization effects could be obtained. Thus, Experiment 2 yields evidence that CA has a causal moderating influence on EC and on generalization effects. In this experiment, generalization worked equally well on the category and on the exemplar level. In Experiment 3, different stimuli were used. Results revealed generalization effects for new exemplars and the category itself in the focus condition. Conversely, in the control and distraction condition (low CA), generalization effects were found on the category but not on the exemplar level. Finally, Experiment 4 yielded a data pattern that is comparable with the previous experiments, and showed that generalization effects can also be obtained with naturalistic stimuli. However, generalization on the GSs was found only when the group-defining feature was present during the evaluation of the GSs, although the evaluation of this feature *per se* was not affected by the conditioning. This supports the assumption that the reported generalization effects were not due to conditioning of the visible category feature.

Although EC is a popular and well-established research paradigm, most EC research so far has focused on changing affective reactions toward single CSs but not on attitude change toward the whole CS category or other stimuli related to the CS. There exist a few studies that already yield first evidence for generalization effects in EC (e.g., Walther, 2002; Olson and Fazio, 2006; Hütter et al., 2013; Spruyt et al., 2014). Our research builds on these findings but also extends the existing literature with regard to at least three aspects:

First, pre-conditioning as described in the spreading attitude effect (Walther, 2002) is only one possibility of establishing an association along which an evaluation can generalize. Our findings show that shared category membership provides an alternative way for establishing an association between CSs and category as well as between CSs and GSs, which then results in generalization from the CSs to other category exemplars and to the category itself.

Second, however, the picture that emerged was more complex. Results revealed that it is important to distinguish between generalization from the CSs to new category members and from the CSs to the whole category. The generalization findings from Experiments 1–4 indicate that generalization works better from CSs to the category than from CSs to other exemplars. On the one hand, we cannot rule out that the occurrence of generalization effects on the category level might partly reflect an EC instead of a generalization effect because the category was already salient during conditioning (except Experiment 1). On the other hand, this finding could indicate that generalization works better from CSs to the category than from CSs to other exemplars. Participants always learned directly to which category a CS belongs. In contrast, CSs and new exemplars were related only indirectly via the shared category membership. According to this reasoning, generalization on the exemplar level should be mediated by the category evaluation. Exploratory mediation analyses of our data support this assumption^3^.

Third, our findings demonstrate that CA is a relevant moderating variable. Specifically, generalization effects were stronger (and partly only present) when participants were aware of the US valence of the respective CS–US pairings. This finding is comparable to EC research demonstrating stronger EC effects given high CA (Hofmann et al., 2010). When EC occurs without CA the underlying process is assumed to be mainly associative (e.g., Walther, 2002; Hütter and Sweldens, 2013). In contrast, EC effects that depend on high CA are assumed to be mainly propositional (e.g., Pleyers et al., 2007; Stahl and Unkelbach, 2009). Our own findings regarding CA indicate that propositional processes are more likely to account for generalization effects. However, the stimulus level of generalization (category vs. exemplar) might also play a role. Across all four experiments, we found no generalization effects on the exemplar level when CA was low. This indicates that generalization on this level is mainly driven by propositional reasoning. However, we still assume that CSs and GSs are associated via common group membership but this association is a not a sufficient precondition for generalization effects at the exemplar level to occur. Conversely, generalization effects on the category level occurred in Experiments 3 and 4 even when participants were low in CA, suggesting that associative processes might also play a role for generalization at the category level. However, this remains speculative until tested directly in future research. An alternative explanation for the finding that category generalization effects in two experiments were not dependent on CA focuses on the specific CA assessment that measured awareness at the item and not at the category level. It is possible that people have encoded only category-valence relations during EC (at least in the *information before EC* conditions) while ignoring other features of the CSs. Asking participants which category was paired with positive images and which with negative images might have led to different levels of CA. Consequently, category-based CA might differ from item-based CA with regard to its influence on generalization at the category level. Future research should therefore assess CA both at the exemplar and the category level.

### Limitations and Future Directions

In sum, our findings are encouraging in that they demonstrate that attitudes toward specific stimuli acquired via EC transfer to other stimuli sharing category membership with the original CS as well as to the category as a whole. The present research also points to new research questions.

First of all, our studies used only fictitious groups, thereby raising the question if our findings also apply to real groups. The primary focus of the present research was to investigate whether attitude transfer effects within EC can generally be obtained. From an ethical point of view, it was more appropriate to use fictitious instead of real social groups because this allowed us to test positive as well as negative transfer effects. Future research, however, needs to extend the investigation of generalization effects within EC into a real-world context (see Olson and Fazio, 2006).

Second, the present findings give first insight into the processes underlying generalization within EC. Despite the debate whether EC is based on associative or propositional processes, there is ample evidence for both. Correspondingly, as indicated by our results and as proposed in the LAC model (Glaser et al., 2015), generalization effects might be also driven by both associative and propositional mechanisms, depending, for instance, on the level at which generalization is assessed (category vs. exemplar). One prominent way of directly approaching the question of underlying mechanisms pertains to the use of direct versus indirect attitude measures (see Olson and Fazio, 2006; Spruyt et al., 2014; Glaser et al., 2015). One shortcoming of the present research is that no indirect attitude measures were used. Future studies should therefore administer direct vs. indirect measures in order to give more insight into the processes underlying generalization within EC.

Taken together, generalization from one attitude object to another one can be the result of EC. Specifically, the present research provides compelling evidence that attitudes toward categories and toward their members can be effectively changed by promoting positive experiences with single category members. Our findings also support the idea that well established effects such as the impact of intergroup contact on attitudes (i.e., contact with one out-group member results in more positive attitudes toward the whole out-group) might be explained with EC mechanisms. Finally, we are optimistic that EC could be a promising strategy to be applied in interventions aimed to reduce prejudice and improve intergroup relations.

## Author Contributions

TG and DK designed and conducted the experiments and analyzed the data. TG wrote the main part of the paper.

## Conflict of Interest Statement

The authors declare that the research was conducted in the absence of any commercial or financial relationships that could be construed as a potential conflict of interest.

## Footnotes

1 We also tested whether the EC effect would be moderated by CA. Only when participants scored high on CA, the EC effect for the CS M–T was found. However, as this was not the focus of the present experiment, we do not report the statistical details.

2 For testing the conditional effects, one SD above the mean of the moderator (which would be the standard procedure) was replaced with the maximum value of 1 because one SD above the mean is outside of the range of the data.

3 For Experiments 1 and 3, we tested in two mediation analyses whether the category evaluation would mediate the generalization effect of EC on the GS evaluation. In Experiment 1, the overall model was significant [*R*^2^ = 0.44, *F*(2, 121) = 48.47, *p* < 0.001]. Importantly, the analysis yielded a significant indirect effect of the valence manipulation on the GS evaluation via the category evaluation (*ab* = 36.47, *SE* = 8.76, LL*CI* = 21.62, UL*CI* = 55.74) and no significant direct effect (c′ = 8.19, *SE* = 9.11, LL*CI* = -9.84, UL*CI* = 26.21). For Experiment 3, we found results similar to Experiment 1 with a significant overall model [*R*^2^ = 0.22, *F*(2, 115) = 16.29, *p* < 0.001], a significant indirect effect (*ab* = -16.36, *SE* = 5.71, LL*CI* = -28.82, UL*CI* = -6.98) and a non-significant direct effect (c′ = 8.56, *SE* = 6.38, LL*CI* = -4.08, UL*CI* = 21.20). Finally, although Experiment 2 did not show differential generalization effects for category vs. exemplar, we also conducted a mediation analysis and found a partial mediation effect. Concretely, results revealed a significant overall model [*R*^2^ = 0.40, *F*(2, 141) = 47.13, *p* < 0.001], a significant indirect effect of the valence manipulation on the GS evaluation via the category evaluation (*ab* = -25.01, *SE* = 10.12, LL*CI* = -47.82, UL*CI* = -8.23) but also a significant direct effect (c′ = -44.53, *SE* = 10.05, LL*CI* = -64.39, UL*CI* = -24.67).
